# Supplementary material for: Vacuum Rabi splitting of a dark plasmonic cavity mode revealed by fast electrons
Source: Nat Commun. 2020 Jan 24;11:487. doi: 10.1038/s41467-020-14364-3 (PMC6981195; doi:10.1038/s41467-020-14364-3)
Supplement: Supplementary file 1 — Supplementary Information [file 41467_2020_14364_MOESM1_ESM.docx]

Supplementary Information

**Vacuum Rabi splitting of a dark plasmonic cavity mode revealed by fast electrons**

Bitton et al.

**
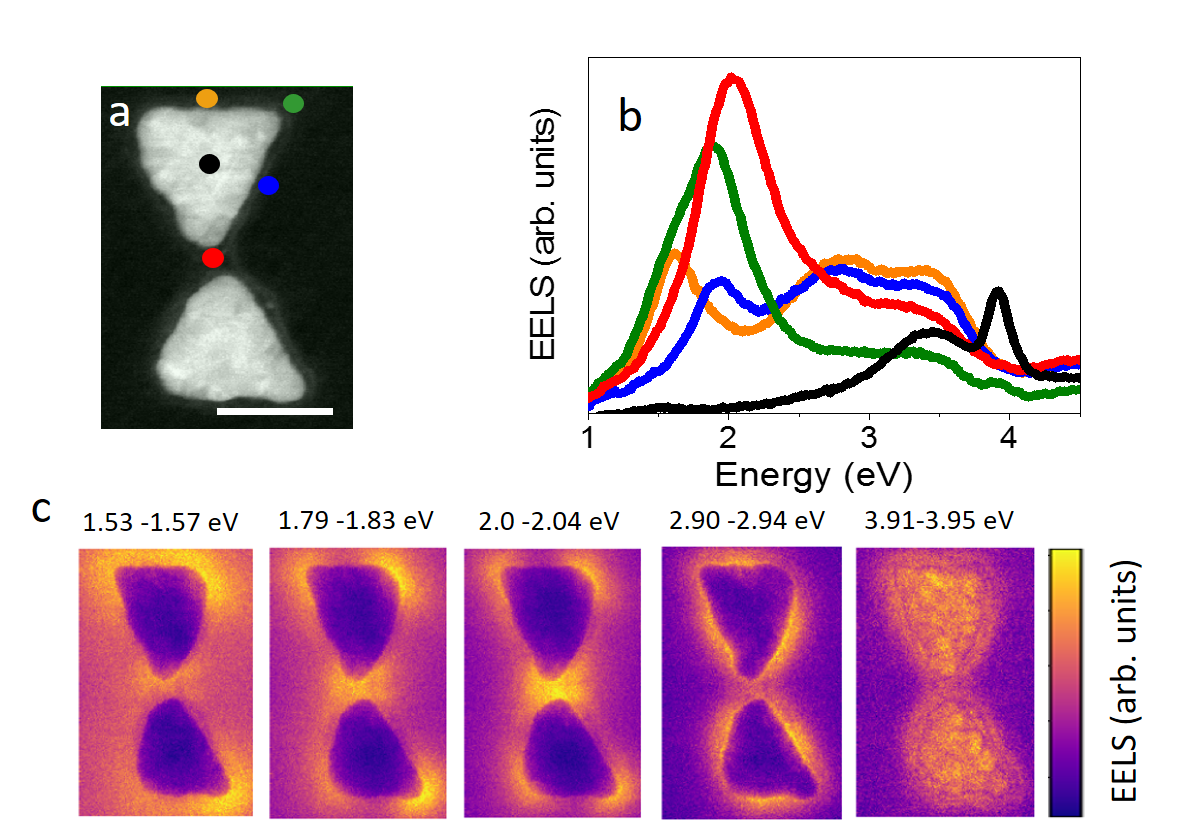
**

**Supplementary Fig 1. EEL spectroscopy of a bare plasmonic bowtie.** (a) STEM image of a bowtie without QDs. Scale bar is 50nm. (b) experimental EEL spectra obtained for different beam locations indicated by matching colored dots in a. (c) Experimental EEL maps for different specified energies ranges, corresponding to the different modes obtained in b.

**
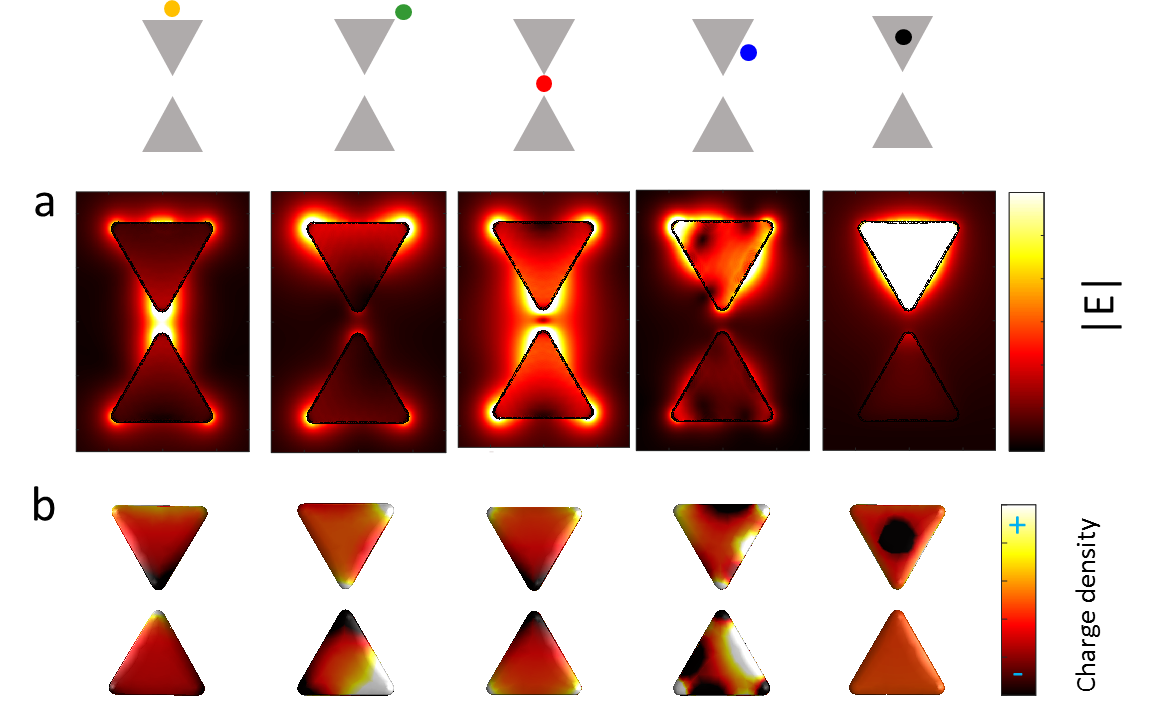
**

**Supplementary Fig 2. Simulated EEL maps for the different modes.** (a) Total electric field distribution projected on the x-y plane. (b) Charge density. All Maps were created for a bare PC at a certain energy and beam location, as specified in the upper part of the figure.

**
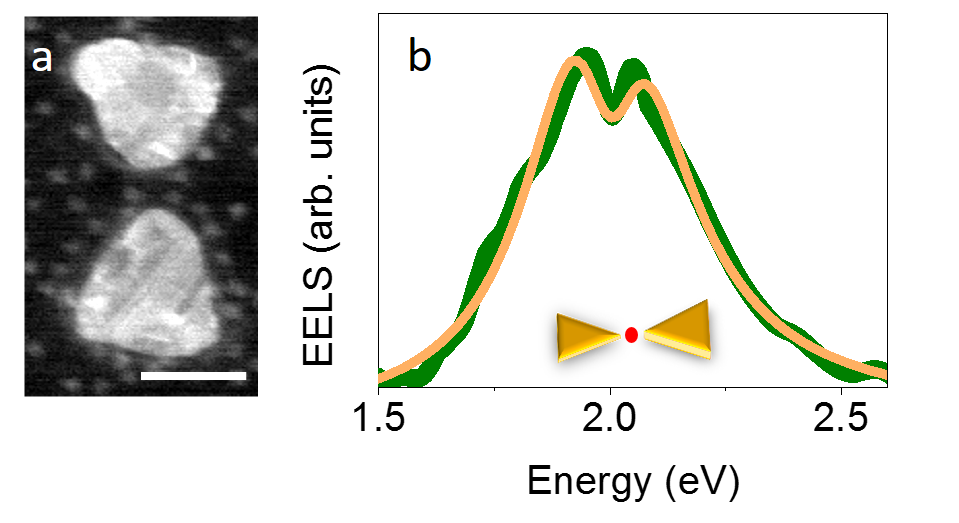
**

**Supplementary Fig. 3. A second example of QDs coupling to a PC at the periphery of the gap.** (a) STEM image of a device loaded with QDs with an exciton energy of 2.0 eV such that all emitters are located away from the center of the gap. Scale bar represents 50 nm. (b) EEL spectrum of the dark dipolar mode of the device in a showing Rabi splitting. The green curve is experimental data, while the orange curve is a fit to the coupled oscillator model, as described in the Methods section. The fit yields a coupling strength of 64±1 meV. Inset demonstrates the point of excitation by the electron beam.

**Supplementary Fig. 4. The continuous nature of Rabi splitting.** Coupled-oscillator model simulations of EELS spectra with increasing coupling strengths, given in the legend in meV. A clear splitting is already seen with a coupling strength of 55 meV, though the two branches of the spectrum split significantly when the second criterion discussed in the text is fulfilled (120 meV). The largest dark-mode coupling strength in our EEL spectra is 85 meV (blue spectrum).
